# Bioevaluation of Novel Anti-Biofilm Coatings Based on PVP/Fe_3_O_4_ Nanostructures and 2-((4-Ethylphenoxy)methyl)-*N*-(arylcarbamothioyl)benzamides

**DOI:** 10.3390/molecules190812011

**Published:** 2014-08-12

**Authors:** Carmen Limban, Alexandru Vasile Missir, Alexandru Mihai Grumezescu, Alexandra Elena Oprea, Valentina Grumezescu, Bogdan Ștefan Vasile, Gabriel Socol, Roxana Trușcă, Miron Teodor Caproiu, Mariana Carmen Chifiriuc, Bianca Gălățeanu, Marieta Costache, Laurențiu Morușciag, Grațiela Pîrcălăbioru, Diana Camelia Nuță

**Affiliations:** 1 Department of Pharmaceutical Chemistry, “Carol Davila” University of Medicine and Pharmacy, Traian Vuia No. 6, 020956 Bucharest, Romania; E-Mails: carmen_limban@yahoo.com (C.L.); missir_alexandru@yahoo.com (A.V.M.); morusciag@yahoo.com (L.M.); diananuta@yahoo.com (D.C.N.); 2 Department of Science and Engineering of Oxide Materials and Nanomaterials, Faculty of Applied Chemistry and Materials Science, University Politehnica of Bucharest, Polizu Street No. 1–7, 011061 Bucharest, Romania; E-Mails: grumezescu@yahoo.com (A.M.G.); elena_oprea_93@yahoo.co.uk (A.E.O.); valentina.grumezescu@inflpr.ro (V.G.); bogdan.vasile@upb.ro (B.Ș.V.); 3 National Institute for Lasers, Plasma & Radiation Physics, Lasers Department, P.O. Box MG-36, Bucharest-Magurele, Romania; E-Mail: gabriel.socol@inflpr.ro; 4 S.C. Metav-CD S.A., 31Rosetti Str., 020015 Bucharest, Romania; E-Mail: roxanatrusca@yahoo.com; 5 The Organic Chemistry Center of Romanian Academy “Costin C.D. Nenitescu” Bucharest, Splaiul Independentei, 202B, 77208 Bucharest, Romania; E-Mail: mtc@cco.ro; 6 Department of Microbiology, Faculty of Biology, University of Bucharest, Research Institute of University of Bucharest, Aleea Portocalelor No. 1–3, 060101 Bucharest, Romania; E-Mail: carmen_balotescu@yahoo.com; 7 Department of Biochemistry and Molecular Biology, University of Bucharest, 91-95 Splaiul Independenței, 050095 Bucharest, Romania; E-Mails: bianca.galateanu@gmail.com (B.G.); marietacostache@yahoo.com (M.C.); 8 Conway Institute, University College Dublin, Dublin 4, Dublin, Ireland

**Keywords:** benzamides, thiourea derivatives, core/shell nanostructure, magnetite, anti-biofilm

## Abstract

Novel derivatives were prepared by reaction of aromatic amines with 2-(4-ethylphenoxymethyl)benzoyl isothiocyanate, affording the *N*-[2-(4-ethylphenoxymethyl)benzoyl]-*N*'-(substituted phenyl)thiourea. Structural elucidation of these compounds was performed by IR, NMR spectroscopy and elemental analysis. The new compounds were used in combination with Fe_3_O_4_ and polyvinylpyrrolidone (PVP) for the coating of medical surfaces. In our experiments, catheter pieces were coated by Matrix Assisted Pulsed Laser Evaporation (MAPLE) technique. The microbial adherence ability was investigated in 6 multi-well plates by using culture based methods. The obtained surfaces were also assessed for their cytotoxicity with respect to osteoblast cells, by using fluorescence microscopy and MTT assay. The prepared surfaces by advanced laser processing inhibited the adherence and biofilm development ability of *Staphylococcus aureus* and *Pseudomonas aeruginosa* tested strains while cytotoxic effects on the 3T3-E1 preosteoblasts embedded in layer shaped alginate hydrogels were not observed. These results suggest that the obtained medical surfaces, based on the novel thiourea derivatives and magnetic nanoparticles with a polymeric shell could represent a promising alternative for the development of new and effective anti-infective strategies.

## 1. Introduction

In the recent years, thiourea derivatives have gained extensive applications in medicine, agriculture, and also as ligands in coordination chemistry [1]. Specialized literature reveals that thiourea derivatives show a broad spectrum of biological activities. The thiourea skeleton can be effectively used to prepare a large number of new compounds with biological activities such as antiviral [2], anticancer [3], anti-inflammatory [4], antimicrobial [5,6], anticonvulsant [7] and anti-helmintic activities [8]. Thiourea derivatives are used as corrosion inhibitors [9] and as intermediates to obtain a great variety of heterocyclic compounds [10]. The crystal X-ray diffraction study of thiourea derivatives allowed a better understanding of the nature of binding of these compounds and a valuable insight into their conformation [11].

Although antibiotics have saved countless millions of lives, over the last decades, the emergence of antimicrobial resistance has limited their efficiency, becoming a serious global health problem that requires the development of new antimicrobial agents effective against pathogenic microorganisms resistant to currently available treatments [12].

Taking into account the drawback of potential drug candidates to reach their targets, the concept of drug delivery, especially by means of nanoscale carriers has become a focus of modern medicine. Iron oxide nanoparticles exhibit plenty of advantages through which they can be recommended for targeted biomedical applications [13,14,15,16,17,18,19,20,21,22].

The purpose of this study was to obtain novel *N*-[2-(4-ethylphenoxymethyl)benzoyl]-*N*'-(substituted phenyl)thiourea derivatives (**1a**–**c**) and to combine them with magnetic nanoparticles in order to obtain polyvinylpyrrolidone (PVP)/Fe_3_O_4_/**1a**–**c** nanostructures in the form of anti-biofilm coatings for medical devices, such as catheters. 

## 2. Results and Discussion

### 2.1. Synthesis of New 2-((4-Ethylphenoxy)methyl)-N-(arylcarbamothioyl)benzamides **1a**–**c**

**Scheme 1:**
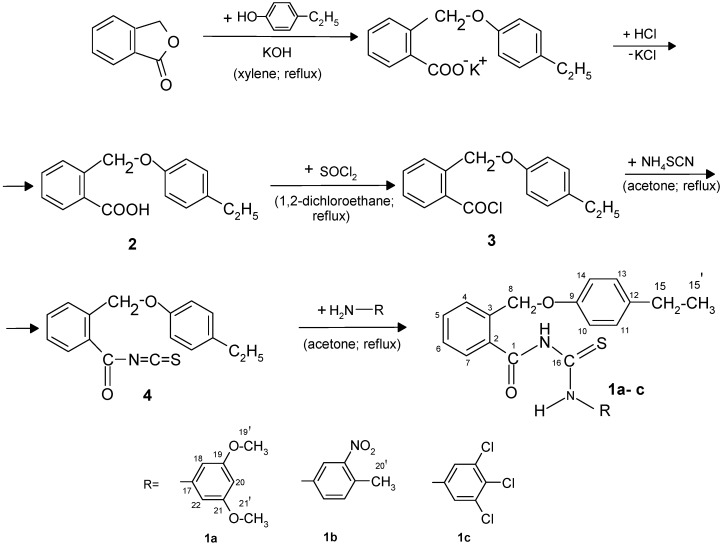
Synthetic pathway for the new *N*-phenylcarbamothioylbenzamides (**1a**–**c**).

The synthetic route to the target compounds, 2-((4-ethylphenoxy)methyl)-*N*-(arylcarbamothioyl)benzamides (**1a**–**c**), is shown in Scheme 1.

The new thioureides are white or light yellow crystalline solids, soluble at room temperature in acetone and chloroform, on heating in lower alcohols, benzene, toluene and xylene but insoluble in water.

The melting points are sharp, indicating the purity of these compounds.

The elemental analyses results were in good agreement with those calculated for the suggested formula, and the accuracy of experimental values in respect to the theoretical values was ±0.4%.

The IR and NMR spectra confirmed the identity of the products while the IR bands were given as w—weak, m—medium, s—strong, vs—very strong. In the IR spectra, some significant stretching bands due to νN-H of amide and thioamide groups and νC=O were observed at 3279–3025 cm^−1^ and 1686–1673 cm^−1^, respectively.

Characteristic for νC-H of methyl and methylene groups, the anti-symmetric stretching vibrations were calculated in the frequency range of 2969–2958 cm^−1^ and respectively, 2930–2929 cm^−1^.

The νC=S stretching vibrations were identified in the range of 1167–1153 cm^−1^ which is in agreement with data reported in the literature [23].

The δN-H amide group stretching band appeared in the region 1511–1508 cm^−1^.

These compounds also show typical alkyl-aryl ether at 1258–1235 cm^−1^, for the anti-symmetric vibration, and 1041–1015 cm^−1^ for the symmetric one.

The structure of compounds is also supported by NMR measurements.

The spectra were registered in hexadeuteriodimethyl sulphoxide (dmso-d6) at 298 K and the chemical shifts values, expressed in parts per million (ppm) were referenced downfield to tetramethylsilane, for ^1^H-NMR and ^13^C-NMR and the constants (*J*) values in Hertz.

The bidimensional correlations spectra (Heteronuclear Multiple Bond Correlation-HMBC, Heteronuclear Single-Quantum Coherence-HSQC and Correlation spectroscopy-COSY spectra) were run for complete attribution of chemical shifts.

In the ^1^H-NMR spectra the apparent resonance multiplicity is described as: s (singlet), d (doublet),t (triplet), q (quartet), m (multiplet), dd (double doublet), td (triple doublet), and br (broad) signal.

The ethyl group exhibited a characteristic quartet at δ 2.51–2.49 ppm and triplet at δ 1.14–1.10 ppm. The methylene group attached to the oxygen atom showed a singlet at δ 5.26–5.27 ppm. 

The NH protons exhibited two characteristic broad singlets or singlets at δ 12.04–11.80 ppm andδ 12.46–12.39 ppm.

For the ^1^H-NMR data the following order is: chemical shifts, multiplicity, the coupling constants, number of protons and signal/atom attribution.

^13^C-NMR signals of the carbonyl group at δ 170.84–169.94 ppm showed the values in the NMR spectra due to the existence of the intra-molecular hydrogen bond related to the carbonyl oxygen atom. Carbon atom of the thiocarbonyl group at δ 179.67–179.28 ppm showed the highest values.

For the ^13^C-NMR data the following order is: chemical shifts and signal/atom attribution (Cq-quaternary carbon) [24].

### 2.2. PVP/Fe_3_O_4_/1a–c Characterization

**Figure 1:**
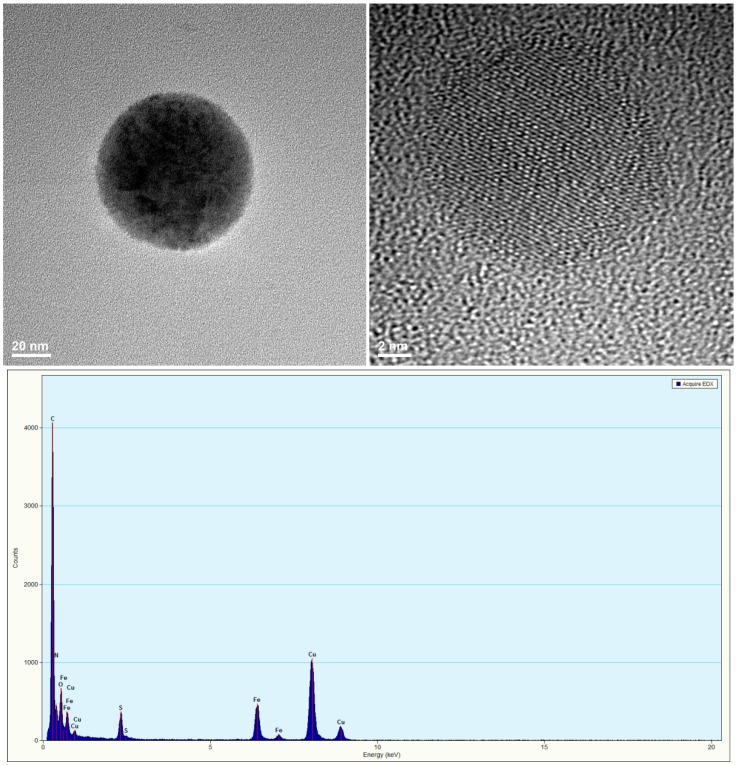
Transmission electron microscopy (TEM) image, HR-TEM image and EDX pattern of PVP/Fe_3_O_4_/**1a**.

Nanostructures, based on PVP, Fe_3_O_4_ and the new compounds **1a**–**c**, were characterized by transmission electron microscopy. The PVP/Fe_3_O_4_/**1a** sample was selected for our discussion, while data for PVP/Fe_3_O_4_/**1b** and PVP/Fe_3_O_4_/**1c** are not shown. The results of TEM analysis are plotted in Figure 1. According to Figure 1a the dimension of prepared PVP/Fe_3_O_4_/**1a** was estimated at 80 nm, while magnetite particles have a diameter around 15 nm (Figure 1b). EDX analysis confirms the presence of Fe, S, N and O as main elements of the analyzed sample.

### 2.3. Thin Films Characterization

#### 2.3.1. IR

**Figure 2:**
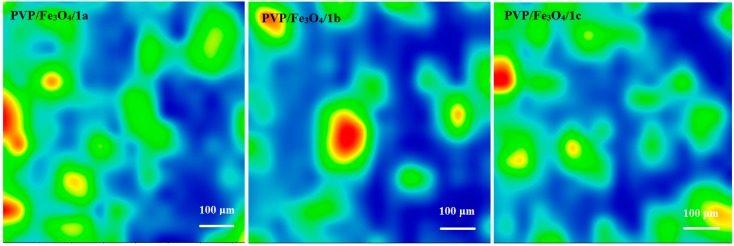
IR mapping of PVP/Fe_3_O_4_/**1a**–**c** dropcast: Intensity distribution of 1639 cm^−1^.

**Figure 3:**
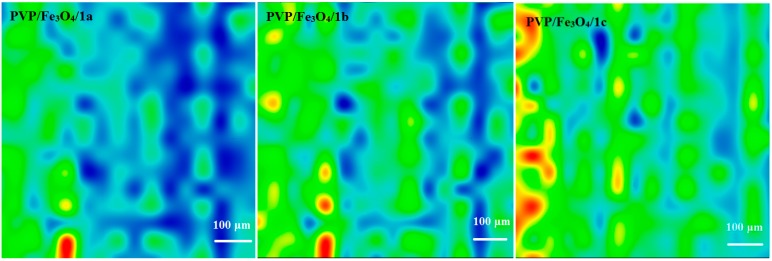
IR mapping of PVP/Fe_3_O_4_/**1a**–**c** thin coatings (F = 400 mJ/cm^2^): Intensity distribution of 1639 cm^−1^.

**Figure 4:**
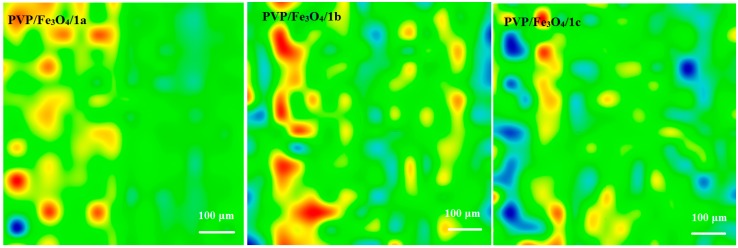
IR mapping of PVP/Fe_3_O_4_/**1a**–**c** thin coatings (F = 500 mJ/cm^2^): Intensity distribution of 1639 cm^−1^.

**Figure 5:**
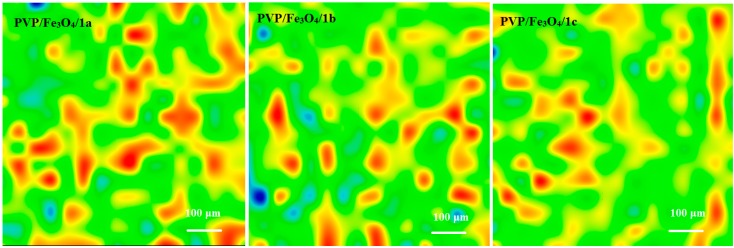
IR mapping of PVP/Fe_3_O_4_/**1a**–**c** thin coatings (F = 600 mJ/cm^2^): Intensity distribution of 1639 cm^−1^.

Figure 2, Figure 3, Figure 4 and Figure 5 show the infrared mappings of PVP/Fe_3_O_4_/**1a**–**c** dropcast and thin coatings. Infrared microscopy allows a non-destructive, quick, easy and reproducible method in order to evaluate the structural integrity of functional groups related to a surface [25]. Absorbance intensities of IR spectra maps are proportional to color changes starting with blue (the lowest intensity) and gradually increasing through green, yellow to finally red (the highest intensity). Approximately 250 spectra were analyzed for each sample [15].

One absorption band known as being characteristic for the PVP was selected as spectral marker for prepared PVP/Fe_3_O_4_/**1a**–**c** (Figure 2, Figure 3, Figure 4 and Figure 5). Selected IR absorption was at 1639 cm^−1^ due to the presence of amide carbonyl groups. 

**Figure 6:**
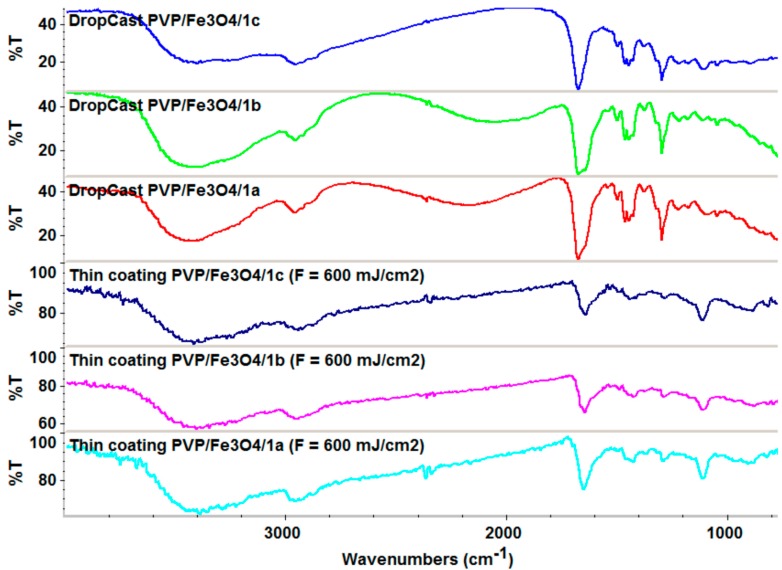
IR spectra of PVP/Fe_3_O_4_/**1a**–**c** dropcast and thin coatings (F = 600 mJ/cm^2^).

Analyzing the intensity distribution of 1639 cm^−1^ on the surface of dropcast it can be concluded that there is no uniformity in this type of coating. By comparing the IR maps from Figure 3, Figure 4 and Figure 5 it can be concluded that thin coatings prepared by MAPLE present a uniform distribution of 1639 cm^−1^ on the entire scanned surface. Also, different intensities of the 1639 cm^−1^ band are observed. Correlated with the IR spectra obtained from IRM analysis (Figure 6), only the thin coating deposited at F = 600 mJ/cm^2^ is suitable for further analyses due to the low degree of degradation of functional groups.

#### 2.3.2. SEM

**Figure 7:**
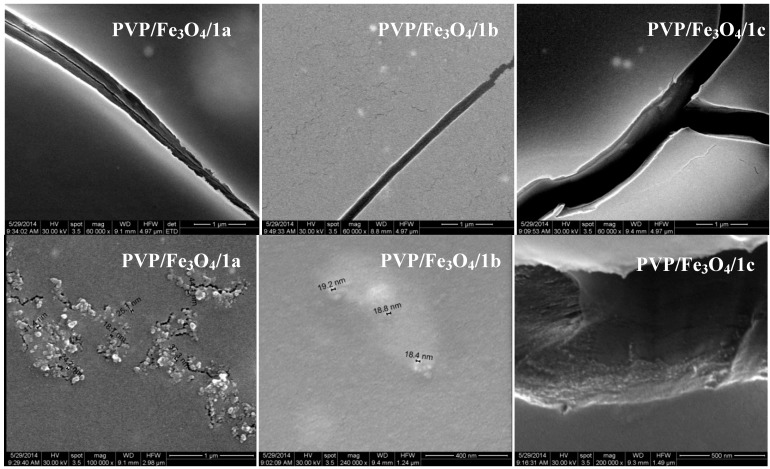
.Scanning electron microscopy (SEM) micrographs of PVP/Fe_3_O_4_/**1a**–**c** thin coatings.

Figure 7 show the SEM micrographs of prepared thin coatings. At 60,000×, it can be observed that the coatings surface is relatively uniform, however at higher magnification (100,000×), aggregates of Fe_3_O_4_ with a diameter between 18–40 nm are observed. Also, some flaws interrupt the pellicle surface.

### 2.4. Biological Assays

#### 2.4.1. Viability and Cell Proliferation

**Figure 8:**
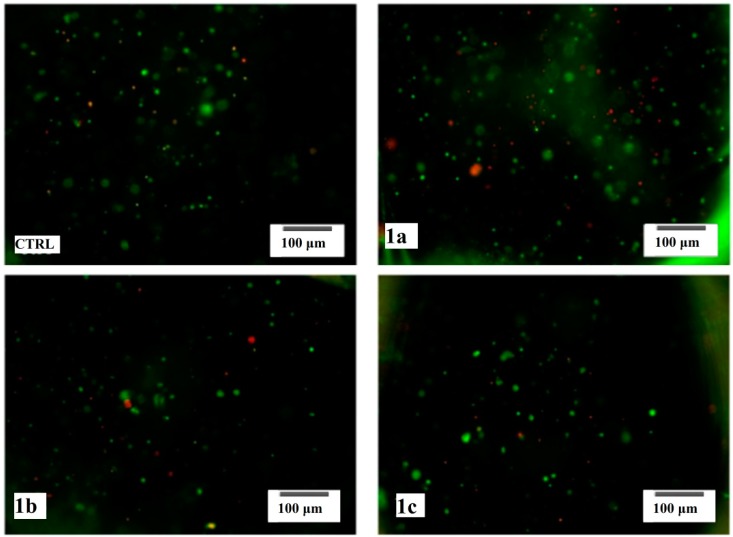
Fluorescence microscopy micrographs of 3T3-E1 preosteoblasts embedded in layer shaped alginate hydrogels, in plain culture medium (control) and exposed to **1a**, **1b** and **1c** compounds, stained with calcein AM (green fluorescence) and ethidium bromide (red fluorescence).

In order to examine the cell survival, the viability of the 3T3-E1 preosteoblasts was assessed after 48 h of exposure to the tested compounds by fluorescence microscopy (Figure 8), based on the simultaneous detection of the live (green labeled) and dead (red labeled) cells inside the 3D hydrogels.

**Figure 9:**
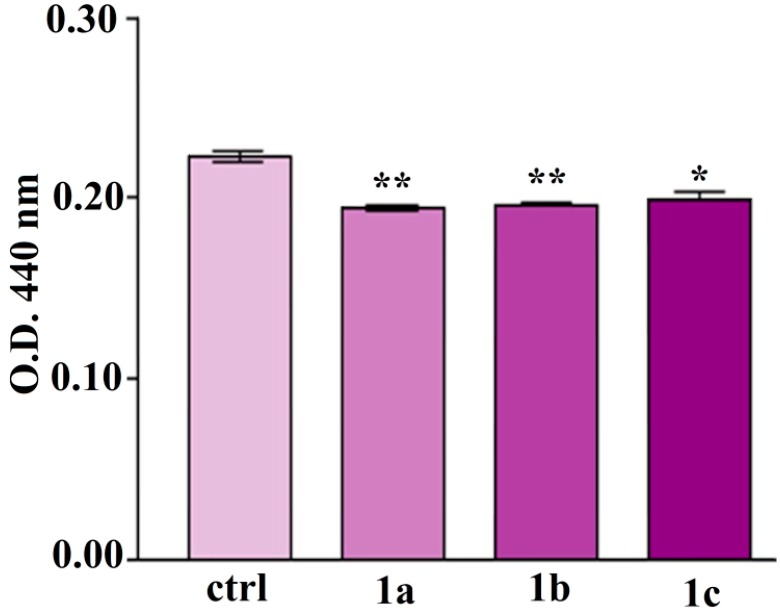
MTT spectrophotometric quantification of 3T3-E1 preosteoblasts viability after 48 h exposure to **1a**, **1b** and **1c**, as compared to the reference (ctrl). (** *p* < 0.001 3T3-E1/AlgH-**1a** bioconstruct *vs.* ctrl, 3T3-E1/AlgH-**1b** bioconstruct *vs.* ctrl, * *p* < 0.013T3-E1/AlgH-**1c** bioconstruct *vs.* ctrl).

As shown in Figure 8, 3T3-E1/AlgH-**1a** displayed a similar cellular density as compared to the 3T3-E1/AlgH control, although the amount of dead cells in these samples was found to be increased in comparison to the reference. These findings could suggest that **1a** does not interfere with cellular proliferation, but could induce an increase of cellular death rate. Interestingly, although the ratio between live and dead cells in 3T3-E1/AlgH-**1b** and 3T3-E1/AlgH-**1c** is positive, the overall cellular density was found to be lower in these samples than in the reference system. These findings could suggest that **1b** and **1c** could inhibit cellular proliferation, but without increasing cellular death rate. To confirm 3T3-E1 preosteoblasts viability observed by fluorescence microscopy, MTT assay was employed as a more accurate approach. In this context, 3T3-E1/AlgH-**1a**, 3T3-E1/AlgH-**1b** and3T3-E1/AlgH-**1c** were subjected to MTT spectrophotometric assay at 48 h of exposure to **1a**, **1b** and **1c** compounds respectively. 3T3-E1/AlgH was subjected to the same procedure at 72 h post seeding (Figure 9).

Our results showed that 3T3-E1 viability after 48 h of exposure to **1a** and **1b** decreased with approx. 12.5% (*p* < 0.001) as compared to the control bioconstruct. The 3T3-E1 preosteoblasts exposed to **1c** for 48 h displayed a lower decrease (approx. 10.7%) in cell viability (*p* < 0.01) as compared to the reference. No significant differences were noticed when comparing the samples.

#### 2.4.2. *In Vitro* Microbial Biofilm Development

Microbial biofilms, defined as sessile microbial communities composed of cells embedded in a extracellular polymeric matrix [26], are highly resistant to limiting environmental conditions and antimicrobial agents, causing chronic, persistent and hard to treat infections [27,28].

The prevention of catheter associated infections could be achieved by using coatings of biomaterials with increased resistance to microbial colonization, by releasing compounds with antimicrobial activity [29,30,31,32]. Hydrophilic polymers such as polyvinylpyrrolidone are used for nanoparticle coatings, liposomes, polymeric micelles, lipoplexes and polyplexes drug carrier systems, in order to prolong bloodstream circulation, acting by reducing opsonization of blood proteins and uptake by macrophages [33].

Taking into consideration the significant antimicrobial activity of thiourea derivatives and using an interdisciplinary approach, we designed a new nanosystem combining new 2-((4-ethylphenoxy)methyl)-*N*-(arylcarbamothioyl)benzamides and a PVP/Fe_3_O_4_ nanostructure in order to obtain a catheter surface coating, with an improved resistance to S. aureus ATCC 25923 and P. aeruginosa ATCC 27853 colonization and subsequent in vitro biofilm development.

**Figure 10:**
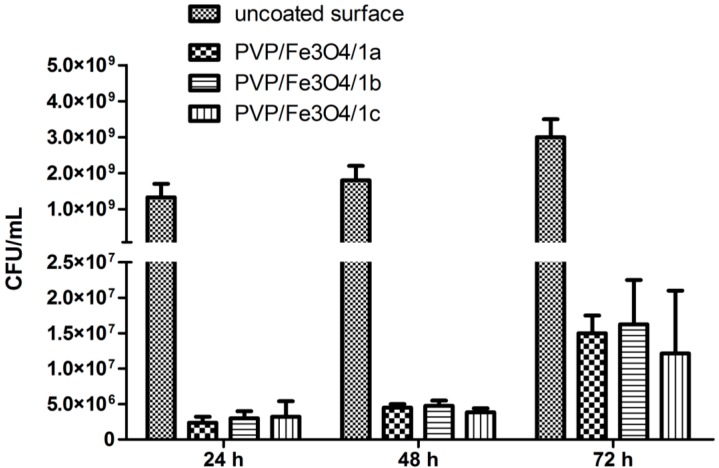
The viable cell counts (VCCs) of *S. aureus* cells embedded in biofilms developed on different catheter sections.

**Figure 11:**
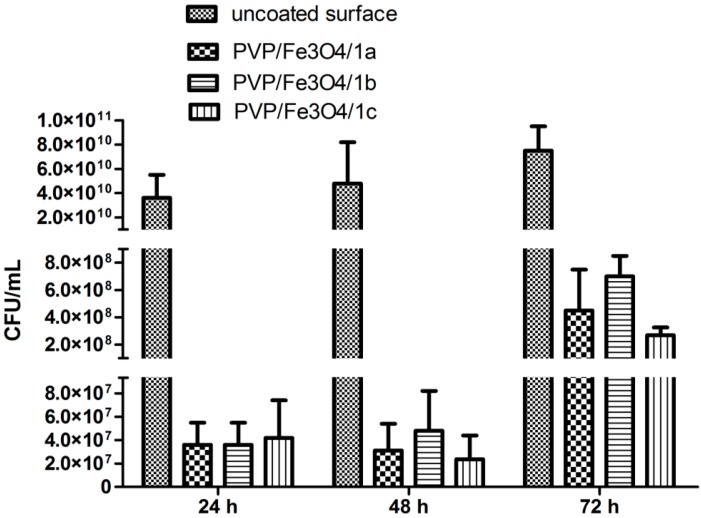
The VCCs of *P. aeruginosa* cells embedded in biofilms developed on different catheter sections.

**Figure 12:**
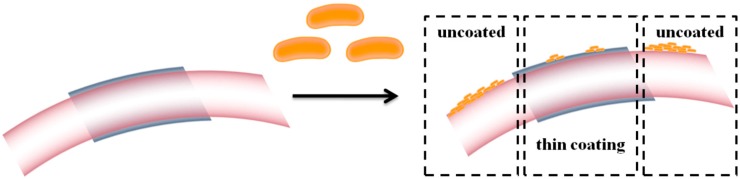
Schematic representation of microbial biofilm formation on the (un)coated surfaces.

In the present study, the development of both *S. aureus* and *P. aeruginosa* biofilms was drastically inhibited on the coated catheter specimens, particularly at 24 h and 48 h (Figure 10, Figure 11 and Figure 12). The behavior of the Gram positive and Gram negative strains was similar, concerning the dynamics of biofilm development in the presence of different substrates.

Overall, the compound 1a (bearing two methoxy groups) exhibited the best anti-biofilm activity at 24 h, thus inhibiting the initial phase of bacterial adherence to the surface, while the compound **1c** (bearing three Cl atoms) proved to be the most active biofilm (quantified at 48 h and 72 h).

## 3. Experimental

### 3.1. Materials

Polyvinylpyrrolidone (PVP), FeCl_3_ (anhydrous, ≥98%), FeSO_4_ (≥99.0%), NH_4_OH (25%) and DMSO were purchased from Sigma-Aldrich. General-use catheter sections, both side polished (100) silicon and glass were provided from a local supplier and used as substrates for the MAPLE coatings.

### 3.2. Preparation of Thiourea Functionalized Magnetite Nanoparticles

New thiourea derivates functionalized magnetite nanoparticles were synthesized according to the previously described protocol [34,35]. Experimentally, FeCl_3_, FeSO_4_ × 7H_2_O (molar ratio 2:1) and 200 mL water were mixed in a 500 mL flask. The prepared iron salt solution was added dropwise into 200 mL of aqueous solution of NH_4_OH, under vigorous stirring. Prepared Fe_3_O_4_ was separated with 100 Kgf NdFeB magnet and washed several times.

**Figure 13:**
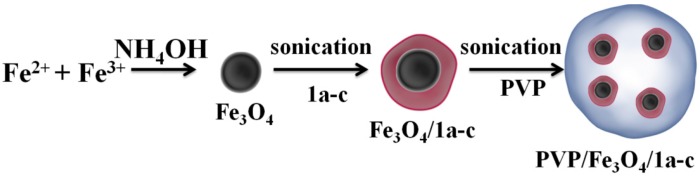
Schematic representation of PVP/Fe_3_O_4_/**1a**–**c** preparation.

The procedure of the PVP/Fe_3_O_4_/**1a**–**c** nanoparticle synthesis is shown in Figure 13. First, in order to prepare Fe_3_O_4_/**1a**–**c**, 5 mL aqueous solution of **1a**–**c** (10%) was added dropwise in 95 mL of as prepared Fe_3_O_4_ solution (1%) under sonication for 30 min. In the next step, the excess of **1a**–**c** was removed by centrifugation at 6,000 rpm for 10 min while the precipitate was redispersed in 10 mL of deionized water. Subsequently, it was added dropwise to 10 mL of PVP (50 mg/mL) aqueous solution under sonication for another 30 min. After this step, the excess PVP was removed by centrifugation at 6,000 rpm for 10 min and the precipitate was redispersed in 20 mL of DMSO for further processing by MAPLE.

### 3.3. MAPLE Thin Coating Deposition

**Figure 14:**
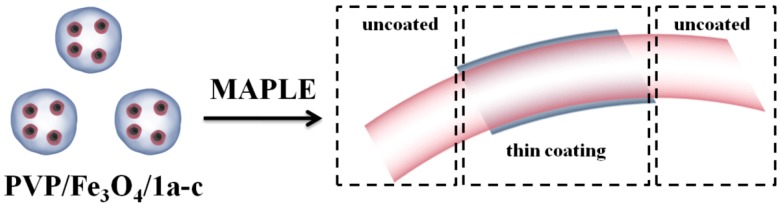
Schematic representation of PVP/Fe_3_O_4_/**1a**–**c** thin coating on the surface of the prosthetic device.

A suspension of 1.5% (w/v) PVP/Fe_3_O_4_/**1a**–**c** in DMSO was prepared. All MAPLE solutions were poured into a pre-cooled target holder and subsequently immersed in liquid nitrogen for 30 min. MAPLE depositions were performed using a KrF* (λ = 248 nm and τ_FWHM_ = 25 ns) laser source COMPexPro 205 model, Lambda Physics-Coherent, that operated at the repetition rate of 10 Hz. The laser fluence was within the range of 400–600 mJ/cm^2^ whereas the laser spot area was set at 36 mm^2^. A laser beam homogenizer was used to improve the energy distribution of the laser spot. In order to avoid the target heating and subsequent drilling, the frozen target was rotated at a rate of 0.4 Hz during coating deposition. All depositions were conducted at room temperature at a background pressure of 1 Pa. All films were grown at a target-substrate separation distance of 4 cm by applying20,000–70,000 subsequent laser pulses. During deposition, the target was kept at a temperature of~173 K by active liquid nitrogen cooling. Thin films were deposited onto catheter sections and both sides polished (100) silicon for IRM, SEM and biological assays. Prior to introduction inside the deposition chamber, the substrates were successively cleaned in an ultrasonic bath with acetone, ethanol and deionized water for 15 min, dried in a jet of high purity nitrogen and then plasma-cleaned in an oxygen atmosphere for 15 min with a plasma system model “FEMTO” from Diener electronic GmbH. During the deposition, the substrates were continuously rotated. Thus, the PVP/Fe_3_O_4_/**1a**–**c** nanosystems (Figure 14) were uniformly spread over the surface of the substrates. For comparison data, a control set of films was prepared by drop casting on the (100) silicon.

### 3.4. Synthesis of 2-((4-Ethylphenoxy)methyl)-N-(arylcarbamothioyl)benzamides ***1a**–**c***

All the reagents and solvents were obtained from commercial sources (Aldrich-Steinheim, Germany; Merck-Darmstadt, Germany) and used as received, except for the solvents which were purified by distillation. Acetone and 1,2-dichloroethane were dried over calcium chloride before use. Ammonium thiocyanate was dried by heating at 100 °C and then used in the reactions.

Melting points were obtained by means of an Electrothermal 9,100 capillary melting point apparatus (Bibby Scientific Ltd, Stone, UK) in open capillary tubes; the values reported herein are uncorrected.

Elemental analysis was performed on a PerkinElmer 2400 Series II CHNS/O Elemental Analyzer (Waltham, MA, USA).

The Fourier-transform infrared (FT-IR) spectra of the all synthesized compounds were performed on a Bruker Vertex 70 FT-IR spectrometer (Bruker Corporation, Billerica, MA, USA).

^1^H-NMR and ^13^C-NMR spectra were recorded on a Bruker Fourier 300 and a Varian 2,000 apparatus, both operated at 300 MHz for ^1^H and 75 MHz for ^13^C, respectively. 

The 2-(4-ethylphenoxymethyl)benzoic acid (**2**) (0.01 mol) was refluxed with thionyl chloride in 1,2-dichloroethane. The isothiocyanation of obtained 2-(4-ethylphenoxymethyl)benzoyl chloride (**3**) (0.01 mol) solubilized in acetone (15 mL) was carried out with ammonium thiocyanate (0.01 mol) in acetone (5 mL) followed by heating for 1 h and then cooling at the room temperature. Arylisothiocyanate (**4**) was coupled with primary amines (0.01 mol) in acetone (2 mL) to give the new compounds (**1a**–**c**) in good yields.

*2-((4-Ethylphenoxy)methyl)-N-(3,5-dimethoxyphenylcarbamothioyl)benzamide* (**1a**). Yield 74%; mp 135–136.7 °C; ^1^H-RMN (dmso-d6, δ ppm): 12.43 (s, 1H, NH, deuterable); 11.80 (br s, 1H, NH, deuterable); 7.61 (br d, *J* = 7.4 Hz, 1H, H-7); 7.58 (dd, *J* = 1.6 Hz, *J* = 7.4 Hz, 1H, H-4); 7.55 (td, *J* = 1.4 Hz, *J* = 7.4 Hz, 1H, H-5); 7.46 (td, *J* = 1.4 Hz, *J* = 7.8 Hz, 1H, H-6); 7.05 (d, *J* = 8.6 Hz, 2H, H-11-13); 6.89 (d, *J* = 8.6 Hz, 2H, H-10-14); 6.88 (d, *J* = 2.2 Hz, 2H, H-18, H-22); 6.42 (t, *J* = 2.2 Hz, 1H, H-20); 5.26 (s, 2H, H-8); 3.75 (s, 6H, H-19' and H-21'); 2.51 (q, *J* = 7.6 Hz, 2H, H-15); 1.12 (t, *J* = 7.6 Hz, 3H, H-15’). ^13^C-RMN (dmso-d6, δ ppm): 179.28 (C-16); 170.84 (C-1); 160.97 (C-19, C-21); 156.97 (C-9); 140.09 (Cq); 136.92 (Cq); 136.48 (Cq); 133.96 (Cq); 131.72 (C-5); 129.28 (C-11, C-13); 129.15 (C-4); 129.06 (C-7); 128.43 (C-6); 115.30 (C-10, C-14); 102.85 (C-18, C-22); 98.93 (C-20); 68.23 (C-8); 56.03 (C-19', C-21'); 27.92 (C-15); 16.37 (C-15'). FT-IR (solid in ATR, ν cm^−1^): 3279 w; 3025 w; 2958 w; 2930 w; 2836 w; 1673 w; 1574 s; 1511 vs; 1456 m; 1360 w; 1328 s; 1235 s; 1196 m; 1153 vs; 1120 m; 1041 m; 892 w; 816 m; 741 w; 684 m; 596 w. Anal. Calcd for C_25_H_26_N_2_O_4_S (450.55): C, 66.65; H, 5.82; N, 6.22; S, 7.12%; Found: C, 66.51; H, 5.89; N, 6.31; S 7.18%.

*2-((4-Ethylphenoxy)methyl)-N-(3-nitro-4-methylphenylcarbamothioyl)benzamide* (**1b**). Yield 81%; mp 126.3–127.6 °C; ^1^H-RMN (dmso-d6, δ ppm): 12.46 (s, 1H, NH, deuterable); 11.97 (br s, 1H, NH, deuterable); 8.38 (d, *J* = 2.2Hz, 1H, H-18); 7.74 (dd, *J* = 2.2 Hz, *J* = 8.2 Hz, 1H, H-22); 7.61 (br d, *J* = 7.4 Hz, 1H, H-7); 7.58 (dd, *J* = 1.6 Hz, *J* = 7.4 Hz, 1H, H-4); 7.55 (td, *J* = 1.4 Hz, *J* = 7.4 Hz, 1H, H-5); 7.54 (d, *J =* 8.2 Hz, 1H, H-21); 7.47 (td, *J =* 1.4 Hz, *J =* 7.8 Hz, 1H, H-6); 7.05 (d, *J =* 8.6 Hz, 2H, H-11-13); 6.88 (d, *J =* 8.6 Hz, 2H, H-10, H-14); 5.27 (s, 2H, H-8); 2.50 (s, 3H, H-20’); 2.49 (q, *J =* 7.6 Hz, 2H, H-15); 1.10 (t, *J =* 7.6 Hz, 3H, H-15). ^13^C-RMN (dmso-d6, δ ppm): 179.56 (C-16); 169.99 (C-1); 156.23 (C-9); 148.16 (C-19); 136.73 (Cq); 136.19 (Cq); 135.79 (Cq); 133.19 (Cq); 132.75 (C-21); 131.00 (C-5); 130.57 (Cq); 129.44 (C-22); 128.52 (C-11, C-13); 128.38 (C-4); 128.31 (C-7); 127.70 (C-6); 120.06 (C-18); 114.50 (C-10, C-14); 67.48 (C-8); 27.18 (C-15); 19.19 (C-20'); 15.63 (C-15'). FT-IR (solid in ATR, ν cm^−1^): 3182 m; 3027 m; 2969 m; 2929 m; 2871 w; 1686 m; 1583 m; 1506 vs; 1451 s; 1379 m; 1337 s; 1258 m; 1213 m; 1155 vs; 1068 m; 1015 m; 880 w; 828 m; 692 m; 657 m; 597 w; 547 w. Anal. Calcd for C_24_H_23_N_3_O_4_S (449.52): C, 64.13; H, 5.16; N, 9.35; S, 7.13%; Found: C, 64.41; H, 5.25; N, 9.49; S 7.14%.

*2-((4-Ethylphenoxy)methyl)-N-(3,4,5-trichlorophenylcarbamothioyl)benzamide* (**1c**). Yield 74%; mp 152–153.2 °C; ^1^H-NMR (dmso-d6, δ ppm): 12.39 (br s, 1H, NH); 12.04 (br s, 1H, NH); 7.92 (s, 2H, H-18, H-22); 7.60 (br d, *J =* 7.4 Hz, 1H, H-7); 7.59 (m, 1H, H-4); 7.57 (td, *J =* 1.4 Hz, *J =* 7.4 Hz, 1H, H-5); 7.46 (td, *J =* 1.4 Hz, *J =* 7.5 Hz, 1H, H-6); 7.08 (d, *J =* 8.6 Hz, 2H, H-11, H-13); 6.89 (d, *J =* 8.6 Hz, 2H, H-10, H-14); 5.26 (s, 2H, H-8); 2.51 (q, *J =* 7.5 Hz, 2H, H-15); 1.14 (t, *J =* 7.5 Hz, 3H, H-15’). ^13^C-NMR (dmso-d6, δ ppm): 179.67 (C-16); 169.94 (C-1); 156.25 (C-9); 137.97; 136.17; 135.85; 133.14; 132.35; 131.07 (C-5); 128.52 (C-11, C-13); 128.38 (C-4); 128.35 (C-7); 127.73 (C-6); 126.85; 125.16 (C-18, C-22); 114.48 (C-10, C-14); 67.45 (C-8); 27.22 (C-15); 15.68 (C-15'). FT-IR (solid in ATR, ν cm^−1^): 3249 w; 3073 w; 2961 w; 1679 m; 1559 m; 1508 vs; 1435 m; 1384 w; 1305 m; 1244 s; 1167 m; 1040 m; 859 w; 817 m; 698 w; 666 m; 613 w. Anal. Calcd for C_23_H_19_Cl_3_N_2_O_2_S (493.83): C, 55.94; H, 3.88; Cl 21.54; N, 5.67; S, 6.49%; Found: C, 55.81; H, 3.97; N, 5.58; S, 6.54%.

### 3.5. Characterization

#### 3.5.1. X-ray Diffraction

X-ray diffraction analysis was performed on a Shimadzu XRD 6,000 diffractometer at room temperature. In all the cases, Cu Kα radiation from a Cu X-ray tube (run at 15 mA and 30 kV) was used. The samples were scanned in the Bragg angle 2θ range of 10–80°.

#### 3.5.2. Infrared Microscopy

IR mapping were recorded on a Nicolet iN10 MX FT-IR Microscope with a MCT liquid nitrogen cooled detector in the measurement range 4,000–725 cm^−1^. Spectral collection was made in reﬂection mode at 4 cm^−1^ resolution. For each spectrum, 32 scans were co-added and converted to absorbance using OmincPicta software (Thermo Scientiﬁc). Approximately 250 spectra were analyzed for each coating and dropcast. Two absorptions peaks known as characteristic for the PVP/Fe_3_O_4_/**1a**–**c** were selected as spectral markers of nanoparticles presence in the prepared coatings.

#### 3.5.3. Scanning Electron Microscopy

Scanning electron microscopy (SEM) analysis was performed on a FEI electron microscope, using secondary electron beams with energies of 30 keV, on samples covered with a thin gold layer.

#### 3.5.4. TransmissionElectronMicroscopy

The transmission electron microscopy (TEM) images were obtained on finely powdered samples using a Tecnai^TM^ G2 F30 S-TWIN high-resolution transmission electron microscope from the FEI Company (Hillsboro, OR, USA) equipped with EDS and SAED. The microscope was operated in transmission mode at 300 kV with TEM point resolution of 2 A° and line resolution of 1 A°. The fine powder was dispersed in pure ethanol and ultrasonicated for 15 min. After that, the diluted sample was distributed onto a holey carbon-coated copper grid and left to dry before TEM analysis.

### 3.6. Biological Characterization

#### 3.6.1. Viability and Cell Proliferation

##### 3.6.1.1. Cell Culture

In this study 3T3-E1 murine pre-osteoblasts cell line was used. To prepare the 3D culture system, 7 × 10^5^ cells/mL were mixed with sterile 1.5% (w/v) low viscosity sodium alginate (Sigma Aldrich, Co.) in saline solution. The cell-alginate suspension was distributed into the wells of a 24-multiwell culture plate (Nunc) and subjected to the cross-linking process as previously described by Galateanu *et al.* (2012) [36]. Briefly, to produce the 3D bioconstructs, sterile discs of filter paper were soaked with the calcium gluconate (CG) solution (Zentiva) and placed above the cell suspension in each well. An equal volume of cross-linking agent was placed above the disk, and allowed to produce 3D alginate hydrogels (AlgH) for 10–15 min in standard conditions of culture. The resulting thin layer 3T3-E1/AlgH were sequentially washed with saline solution and placed in DMEM culture medium (Sigma Aldrich, Co.) supplemented with 10% fetal bovine serum (FBS) and 1% antibiotic/antimycotic. After 24 h of culture, the culture medium was supplemented with the compounds **1a**, **1b** or **1c** at a final concentration of 1 mg/mL. The reference 3T3-E1/AlgH bioconstruct was not exposed to any of the tested compounds during the experimental period and was maintained in plain culture medium.

For further simplicity, the following abridgements are introduced to designate the studied 3D bioconstructs: 3T3-E1 cells embedded in AlgH and exposed to DMEM culture medium supplemented with 1 mg/mL: (i) 3T3-E1/AlgH-**1a**; (ii) 3T3-E1/AlgH-**1b**; and (iii) 3T3-E1/AlgH-**1c**. The control bioconstruct was maintained in plain culture medium during the experimental time and is further referred to as 3T3-E1/AlgH.

##### 3.6.1.2. Cell Viability

3T3-E1/AlgH-**1a**, 3T3-E1/AlgH-**1b** and 3T3-E1/AlgH-**1c** bioconstructs were subjected to cellular viability tests after 48 h of exposure to **1a**, **1b** and respectively **1c** compounds, as compared to the 3T3-E1/AlgH reference system.

*Live/Dead Fluorescence Microscopy Assay.* The viability of 3T3-E1 cells within 3T3-E1/AlgH-**1a**, 3T3-E1/AlgH-**1b**, 3T3-E1/AlgH-**1c** and 3T3-E1/AlgH bioconstructs was evaluated by fluorescence microscopy using Live/Dead Kit (Invitrogen, Life Technologies, Foster City, CA, USA). This method allows the simultaneous detection of both live and dead cells with calcein acetoxymethyl (calcein AM) and ethidium bromide dyes provided in the kit. Calcein AM is a non-fluorescent and permeable reagent, which is converted by the intracellular esterases to the intensely green fluorescent calcein (ex/em: ~495 nm/~515 nm). Ethidium bromide enters the cells with damaged membranes, producing a bright red fluorescence when binding to nucleic acids (ex/em: ~495 nm/~635 nm). Briefly, at 48 h post seeding, all the bioconstructs were incubated with a staining solution prepared according to the manufacturer’s instructions for 15 min. Next, the stained 3D cultures were analyzed by fluorescence microscopy using an Olympus IX71 inverted microscope, and images were captured with Cell F Imaging Software (Olympus, Hamburg, Germany, 2008).

*MTT Spectrophotometric Test.* The viability of the 3T3-E1 cells within the alginate hydrogels was quantitatively assessed by MTT assay at 48 h post exposure to 1 mg/mL of the **1a**, **1b** and **1c** compounds. This test is based on the reduction of a tetrazolium salt solution (MTT) to purple formazan by metabolically active cells. All the three samples were incubated for 2 h in 1 mg/mL MTT solution (Sigma Aldrich Co., Steinheim, Germany). The concentration of the formazan produced by the metabolically active cells was spectrophotometrically quantified at 550 nm (Appliskan Thermo Scientific, Waltham, MA, USA), after solubilization in DMSO. The result was a sensitive assay with a colorimetric signal proportional to the viable cell number.

#### 3.6.2. *In Vitro* Microbial Biofilm Development

*Staphylococcus aureus* ATCC 25923 and *Pseudomonas aeruginosa* ATCC 27853 strains were purchased from American Type Cell Collection (ATCC). For assessing the biofilm formation, fresh bacteria cultures were obtained in Luria Broth and diluted as mentioned below.

Biofilm formation was assessed using 6 multi-well plates (Nunc), in a static model for monospecific biofilm development. Coated and uncoated substrates (*i.e.*, 0.5 cm samples of uncoated and coated catheters) were distributed in the plates containing 2 mL of microbial inoculum diluted to 10^4^–10^5^ CFU/mL in Luria Broth. Samples were incubated for 24 h at 37 °C. 

After 24 h incubation time, the culture medium was removed and the samples were washed with sterile PBS, in order to remove the unattached bacteria. Coated and uncoated substrates were placed in fresh medium and incubated for additional 24 h, 48 h and 72 h. After the incubation time, the samples were gently washed with sterile PBS to remove the non-adherent cells and placed in 1.5 mL micro-centrifuge tubes (Eppendorf) containing 750 μL PBS. In order to disperse biofilm cells into the suspension, the samples were vigorously mixed by vortexing for 30 s and sonicated for 10 s. Serial ten-fold dilutions were achieved and plated on LB Agar for viable cell counts (VCC) [37]. Experiments were performed in triplicate and repeated on three separate occasions [38].

#### 3.6.3. Statistical Analysis

The statistical significance of the obtained results was analyzed using GraphPad Prism version 5.04 for Windows, GraphPadSoftware, San Diego, CA, USA. For comparison, we used the number of CFU (colony forming units) mL^−1^ as revealed by the readings of three values/experimental variants. Two-way ANOVA and Tukey’s multiple comparison tests were used for revealing significant differences among the analyzed groups.

## 4. Conclusions

New thiourea derivatives were synthesized by the reaction between 2-(4-ethylphenoxymethyl)benzoyl isothiocyanate obtained *in situ* and aromatic amines. The novel compounds were characterized using IR, NMR and elemental analysis and evaluated for their cytotoxicicity on 3T3-E1 preosteoblasts. The results of cellular viability and proliferation demonstrated that the compounds **1a**, **1b** and **1c** did not display cytotoxic effects on the cells embedded in layer shaped alginate hydrogels and therefore they could be used for developing biomedical applications. The incorporation of the novel **1a**, **1b** and **1c** compounds in a core-shell nanosystem represented by magnetic nanoparticles with polymeric shell and the pelliculisation of the obtained nanofluid on catheter samples led to the achievement of an optimized anti-biofilm coating, efficient against *S. aureus* and *P. aeruginosa* biofilms, both in the early and maturation phase. Taken together, these results suggest that the obtained nano-coatings containing the novel thiourea derivatives could represent a promising alternative for the development of modified surfaces with increased resistance to bacterial adherence and biofilm development.
